# Effects of the Chemical Composition on the Antioxidant and Sensory Characteristics and Oxidative Stability of Cold-Pressed Black Cumin Oils

**DOI:** 10.3390/antiox11081556

**Published:** 2022-08-11

**Authors:** Aleksandra Szydłowska-Czerniak, Monika Momot, Barbara Stawicka, Dobrochna Rabiej-Kozioł

**Affiliations:** 1 Department of Analytical Chemistry and Applied Spectroscopy, Faculty of Chemistry, Nicolaus Copernicus University in Toruń, Gagarina 7, 87-100 Toruń, Poland; 2 Bunge Polska Sp. z o.o., Niepodległości 42, 88-150 Kruszwica, Poland

**Keywords:** cold-pressed black cumin oils, antioxidant capacity, tocopherols, sterols, oxidative stability, fatty acid profile, quantitative descriptive sensory analysis, principal component analysis

## Abstract

The antioxidant capacity (AC); amounts of tocopherols, sterols, and polycyclic aromatic hydrocarbons; oxidative parameters; fatty acid composition (FAC); and sensory quality of cold-pressed black cumin oils (CPBCOs) available on the Polish market were analyzed and compared. The AC levels of the CPBCO samples were determined using four assays, namely 2,2-diphenyl-1-picrylhydrazyl (DPPH = 226.8–790.1 μmol TE/100 g), 2,2′-azino-bis(3-ethylbenzothiazoline-6-sulfonic acid) (ABTS = 385.9–1465.0 μmol TE/100 g), cupric-reducing antioxidant capacity (CUPRAC = 975.3–19,823.3 μmol TE/100 g), and Folin–Ciocalteu assays (FC = 168.1–643.7 μmol TE/100 g). The FAC scores were typical for black cumin oil, except for the sample CPBCO4, which had a higher content of α-linolenic acid (C18:3 = 23.33%), pointing to possible oil adulteration. Additionally, the concentrations of total sterols (TSC = 372 mg/100 g) and tocopherols (TTC = 42.3 mg/100 g) in this sample were higher than those for other investigated oils (TSC = 159–222 mg/100 g, TTC = 1.9–10.4 mg/100 g respectively). The oxidative stability levels (IP = 8.21–37.34 h), peroxide values (PV = 21.36–123.77 meq O_2_/kg), acid values (AV = 6.40–22.02 mg KOH/kg), and the sums of four specific polycyclic aromatic hydrocarbons (∑4PAHs = 4.48–46.68 μg/kg) in the studied samples differed significantly (*p* < 0.05). A sensory lexicon including 12 attributes was developed and applied for the sensory evaluation of oils using a quantitative descriptive analysis (QDA).

## 1. Introduction

Cold-pressed vegetable oils are gaining increasing interest among conscious consumers. Nowadays, with health and nutrition becoming so important, virgin oils are being chosen instead of solvent-extracted and refined oils [1]. Recently, extra virgin olive oil, linseed oil, rapeseed oil, and other oils have been widely used in food, cosmetic, and pharmaceutical applications. It is well known that cold pressing is a mechanical process in which the oil is extracted and separated from the seeds without chemical intervention or the addition of any other substances at a temperature that does not exceed 35 °C. Therefore, cold-pressed oils are recognized as natural, healthy, and abundant in nutritious compounds such as tocopherols, polyphenols, and essential fatty acids [2]. *Nigella sativa* L. seeds and cold-pressed black cumin oils (CPBCOs) are part of the diet in Asia and Africa (Egypt, Tunisia). Lately, CPBCOs have gained more extensive popularity in Europe due to their beneficial fatty acid composition (FAC) and bioactive compounds, including tocopherols, sterols, phenolic compounds, and essential oils. These phytochemicals have anti-inflammatory activity and play key roles in supporting the immune system and in the treatment of allergies and atopic dermatitis. Black cumin oils are also valued for their specific taste, and they are suitable for food seasoning [2,3,4].

It is known that *Nigella sativa* L. seeds can consist of 30–50% fat, depending on the cultivar and seed maturity [4,5]. Unsaturated fatty acids constitute 80–90% of the total lipids (predominantly linoleic and oleic acids), making black cumin oil a healthy product. There can be differences between the profiles of fatty acids due to the country of origin; for example, the linoleic acid amounts varied from 37 to 71% in black cumin seeds cultivated in Bangladesh, Egypt, Iran, Morocco, Syria, Tunisia, and Turkey [4,5,6,7].

Besides the favorable fatty acid composition, CPBCOs are rich in bioactive compounds such as tocopherols, dominated by γ- and α-tocopherols, as well as phytosterols, such as β-sitosterol, stigmasterol, and campesterol [3,6,8]. The phenolic compounds such as thymoquinone and carvacrol are responsible for the antiradical activity of this oil [8,9,10]. These chemical compounds are the main terpenes in essential oils from *Nigella sativa* L. seeds, which on the one hand give the oil its antioxidant potential, and on the hand provide a unique sensory profile that makes the black cumin oil very aromatic. Moreover, *trans*-anethole, p-cymene, thymoquinone, and other volatile compounds with a distinctive aroma and taste were identified in essential oils from black cumin seeds [2,3]. On the other hand, various factors such as the seed varieties, oxidation processes, levels of contaminants, storage conditions, and technological processes can affect the sensory profiles of oils from black cumin seeds. The presence of essential oils and tocopherols results in powerful protection against oxidation—much higher than for other cold-pressed oils. The oxidative stability levels of CPBCOs measured as induction period values (IP = 13.45–38.22 h) using the Rancimat test were about 2–10 times higher than the IP values (3.67–13.98 h) for linseed and rapeseed oils but similar to the IP values of roasted pumpkin seed oils (IP = 20.29–34.39 h) [11].

According to the European Commission, *Nigella sativa* L. seeds and black cumin oils are considered as novel foods listed in the Novel Food Catalogue [12], because these products have just started expanding in Eastern and Western Europe. It is known that during the development process, the creation of new products is imperative to optimize parameters such as the shape, color, appearance, odor, taste, consistency, and safety and to encourage the consumer to purchase. Moreover, the interaction of all these components is critical to achieving a complete balance, leading to excellent quality and good acceptability. This approach is required due to consumers demanding food products with high-quality characteristics, while the food industry should find the right way to satisfy the consumers’ requirements. A sensory analysis allows the estimation of the consumer acceptability and quality of food products, acting as an inherent part of the plan to create new products. The quantitative descriptive analysis (QDA) approach has been recognized as a tool for measuring and optimizing the sensory attributes of various food products. Sensory attribute lexicons for the evaluation of cold-pressed sunflower and rapeseed oils using the QDA methodology have been developed and implemented [13,14,15]. Nevertheless, to the best of our knowledge, a QDA methodology to develop the descriptive terminology and sensory profiles of CPBCO samples has not been proposed.

Although information is available on the chemical composition and oxidative stability of black cumin oils, practically nothing is known about the sensory attributes of oils extracted from *Nigella sativa* L. seeds using various techniques. Therefore, the primary aim of this study was to investigate the chemical composition—mainly via the antioxidant compound contents and oxidative stability levels—of CPBCOs available on the Polish market and to correlate them with sensory attributes. A product-specific glossary was prepared, and a QDA was applied to determine and quantify sensorial descriptors of CPBCOs. Moreover, a principal component analysis (PCA) was applied to classify and discriminate oil samples based on their antioxidant and fatty acids profiles, antioxidant potential, oxidative stability, and sensory characteristics.

## 2. Materials and Methods

### 2.1. Chemicals

All reagents of analytical or HPLC grade were purchased from Merck Sp. z o. o. (Warszawa, Poland). Redistilled water was used for the preparation of the solutions.

### 2.2. Materials

The experimental materials consisted of seven commercial cold-pressed black cumin oils originally packed into amber glass bottles (250–500 mL) purchased in shops on the Polish market. Samples were collected randomly from seven well-known Polish manufacturers who pressed black cumin oils from seeds cultivated in different localities of Poland, Egypt, and India. To maintain the discretion of the manufacturers, the oil samples were designated as CPBCO1–CPBCO7. All oil samples were within their stated shelf lives and stored in a refrigerator until analysis.

### 2.3. Determination of Tocopherols and Sterols Contents

The tocopherols were analyzed following ISO 9936:2016 standard [16] using high-performance liquid chromatography with some modifications. Briefly, each oil (0.5 g) was dissolved in hexane (5 mL), injected (5–20 μL) into a LiChrospher 100 Diol (125 × 4 mm, 5 μm particle size) column (Merck, Darmstadt, Germany), and analyzed using an Agilent 1100 HPLC system (Agilent Technologies, Palo Alto, CA, USA) with an autosampler and fluorescence detector. The mobile phase was hexane with tetrahydrofuran (96:4, *v*/*v*%) at a flow rate of 0.8 mL/min. Excitation and emission wavelengths at 280 and 340 nm, respectively, were used.

The sterols were measured using gas chromatography according to ISO 12228-1:2014 [17]. In brief, the oil samples were saponified using a 1 mol/L methanolic potassium hydroxide solution. The sterol fraction was extracted three times with a hexane/methyl tert-butyl ether (1:1) mixture. The separation and quantification of the silylated sterol fraction were performed using capillary gas chromatography (Agilent Technologies 6890, Wilmington, DE, USA) using a DB-35MS (J&W Scientific, Folsom, CA, USA) capillary column (25 m, 0.20 mm i.d. and 0.33 μm film thickness) and a flame ionization detector (FID). The detector and injector temperature was 300 °C, while hydrogen was used as a carrier gas with a 1.5 mL/min flow rate. Here, 5α-cholestane was used as an internal standard and individual sterols in oils were determined depending on their relative retention times according to the standards.

The total sum of the tocopherols and sterols and their percentage distribution were calculated.

### 2.4. Determination of Antioxidant Capacity

Before the antioxidant capacity (AC) determination, methanolic extracts of the studied oils were prepared according to the extraction procedure used for phenolic antioxidants from cold-pressed black cumin seed oil described by Lutterodt et al. [9] with minor modifications. Briefly, 2 g of oil was weighed into test tubes and extracted with 5 mL of methanol for 30 min using an orbital shaker (SHKA25081 CE, Labo Plus, Warszawa, Poland). Then, the test tubes were placed in a refrigerator for 30 min, and the methanolic extracts were separated from the oil samples. The extracted procedure was repeated three times. The methanolic extracts were collected and stored in glass bottles. The extracts were kept in a cold dark place until analysis.

The AC levels of the CPBCOs were analyzed using four modified spectrophotometric methods, namely 2,2-diphenyl-1-picrylhydrazyl (DPPH), 2,2′-azino-bis(3-ethylbenzothiazoline-6-sulfonic acid) (ABTS), cupric-reducing antioxidant capacity (CUPRAC), and Folin–Ciocalteu (FC) methods, as described previously [18].

DPPH method: The 0.5 mL of DPPH solution in methanol (304.0 μmol/L) was added to 0.1–0.2 mL of methanolic oil extracts diluted by methanol to 1.4–1.3 mL, respectively. The obtained mixtures were shaken vigorously and allowed to stand at room temperature in darkness for 15 min. Then, the absorbance of each solution was measured at 517 nm against a reagent blank (0.5 mL of DPPH methanolic solution mixed with 2 mL of methanol).

ABTS method: To generate the ABTS^•+^ solution, potassium persulfate (2.45 mmol/L) and ABTS (7 mmol/L) were mixed (1:0.5) and kept for 16 h in dark conditions. After this, the initial absorbance ABTS^•+^ solution was adjusted to 0.70 at 734 nm through dilution with ethanol. Next, 0.05–0.10 mL of each methanolic oil extract was allowed to react with the ABTS^•+^ solution (2.45–2.40 mL). After 5 min incubation at 30 °C, the absorbance of the reaction mixture was measured at 734 nm against a reagent blank (2.5 mL of ABTS^•+^ solution).

CUPRAC method: Firstly, 2 mL of CuCl_2_ (0.01 mol/L), 2 mL of neocuproine solution (0.0075 mol/L), and 2 mL of ammonium acetate buffer (1 mol/L, pH = 7.0) were added into a 10 mL volumetric flask. Then, 0.1–0.2 mL of oil methanolic extracts were mixed, and the total volume was brought up to 10 mL with redistilled water. The mixture absorbance was recorded against a blank (all reagents without oil extracts) at 450 nm after 30 min incubation at room temperature.

FC method: In a 10 mL volumetric flask, 1.0 mL of methanolic oil extract and 0.5 mL of FC reagent were combined and mixed until homogeneous at room temperature for 3 min. Next, the reaction was neutralized with 1 mL of Na_2_CO_3_ (22.0%) made up to the mark with redistilled water and then allowed to react in darkness for 1 h. The absorbance measurements of the blue-colored solutions were carried out at 765 nm against a reagent blank.

The absorbance of each reaction mixture in a 1 cm glass cell was read with a Hitachi U-2900 spectrophotometer (Hitachi, Tokyo, Japan). The AC levels of methanolic extracts are expressed as micromoles of Trolox equivalents (TE) per 100 g sample.

### 2.5. Determination of Oxidative Stability

The oxidative stability index values of the analyzed oils were determined using a 743 Rancimat instrument made by Metrohm, applying the AOCS Official Method Cd 12b-92 [19]. Here, 3 g of each CPBCO was measured in the Rancimat test instrument at a temperature of 100 °C ± 0.3 °C with a gas flow rate of 20 L/h and 60 mL of water in the measuring vessel.

The peroxide value (PV) of each oil was measured according to ISO 27107 (2010) [20] via potentiometric titration with the use of a 905 Titrando high-end titrator from Metrohm (Warszawa, Poland) after dissolving the oil in the chloroform–glacial acetic acid mixture in the presence of a saturated solution of potassium iodide. The iodide was then oxidized using oil peroxides, and the formed iodine was titrated with sodium thiosulfate to an equivalence point. The result was reported in milliequivalents of oxygen per kilogram of oil (meq O_2_/kg). However, the acid value (AV) was analyzed according to ISO 660 (1996) [21] via the directly acid–base titration of oil in an alcoholic medium against standard potassium hydroxide. The AV is defined as the number of milligrams of potassium hydroxide required to neutralize the free fatty acids (FFA) present in one gram of oil.

### 2.6. Determination of Water and Volatile Matter Content

The water and volatile matter contents in the studied cold-pressed black cumin oils were measured gravimetrically according to ISO 662 (2016) [22]. The amounts of both the moisture and volatile matter were determined in triplicate by drying 10 g of each oil in an oven at the temperature of 103 °C. The drying process was repeated until reaching a constant weight.

### 2.7. Determination of Polycyclic Aromatic Hydrocarbons

Four polycyclic aromatic hydrocarbons (PAHs) limited by EU regulations (benzo(a)pyrene (B(a)P), chrysene (Chry), benzo(a)anthracene (B(a)A), and benzo(b)fluoranthene (B(b)F)) in the analyzed CPBCO samples were determined according to the internal method using high-performance liquid chromatography with a fluorescence detector (HPLC-FLD, Shimadzu, Kyoto, Japan), Zorbax Eclipse PAH column (particle size 3.5 μm, length 150 mm, diameter 4.60 mm, Agilent Technologies, Santa Clara, CA, USA), and Eclipse XDB-C18 precolumn (3.5 μm, 4.6 × 150 mm, Agilent Technologies) at an oven temperature of 30 °C. The oil samples were dissolved in cyclohexane and extracted to dimethyl formaldehyde. Benzo(b)chrysene was used as an internal standard. The calibration standards were prepared via the dilution of the PAH standards into acetonitrile. These four compounds were quantified using calibration curves plotted for each PAH in the concentration range of 0.25–8.50 μg/kg. The limit of detection (LOD) values (0.08, 0.06, 0.04, and 0.07 μg/kg) and limit of quantification (LOQ) values (0.26, 0.20, 0.14, and 0.23 μg/kg) were calculated for B(a)P, Chry, B(a)A, and B(b)F, respectively. The LOD is the lowest concentration of each PAH in the studied oils that can be detected by the proposed method, while the LOQ is the lowest concentration at which an analyte can be quantitated with a linear response and acceptable precision and accuracy.

### 2.8. Determination of Fatty Acid Composition

The fatty acid profile of each CPBCO was determined according to the official method ISO 5508 [23]. Fatty acid methyl esters prepared using the ISO 5509 standard [24] method were separated on a gas chromatograph (HP 5890 GC) equipped with a flame ionization detector (FID) (Hewlett-Packard, Avondale, PA, USA) and a high polar capillary column BPX 70 (60 m × 0.25 mm, 0.25 μm). The temperatures on the injector and detector were adjusted to 250 °C, while the oven temperature was as follows: heating from 150 to 210 °C at 1.3 °C/min, holding at 210 °C for 5 min. The carrier gas was helium at a flow rate of 0.6 mL/min. The identification of fatty acids was accomplished using external fatty acid methyl ester (FAME) standards, and the results are presented as weight percentages of the total fatty acids.

### 2.9. Sensory Evaluation and Quality Assessment

Two sensory leaders with experience in the sensory field of cold-pressed oils sensory evaluations screened differences in the studied samples and reviewed the previous literature in order to identify a sensory flavor lexicon for the CPBCOs [25,26]. The initial list of attributes was presented during the sensory training session (6 sessions of 45 min) with reference samples and definitions. The selection of attributes was conducted according to ISO 11035 (1994) [27]. As can be seen in Table 1, the final lexicon for CPBCOs consisted of 12 attributes, including 2 basic tastes (sweet, bitter), 6 flavors (overall flavor intensity (OFI), herb-like, flower-like, medical-like, fuel-like, spicy-like), 3 mouth feel sensory terms (astringency, pungency, painty), and the color intensity.

The expert panel consisted of ten (three males and seven females, ages 22–52 years), well-trained individuals (100 h of training) experienced in the sensory evaluation of cold-pressed oils. The selection, training, and monitoring of the assessors were conducted according to ISO 8586:1 [28] and ISO 8586:2 [29].

The CPBCO samples were evaluated in triplicate by each panelist in a randomized, balanced black design. The panelists were instructed to assess the intensity of each of the attributes using a 10-cm intensity scale with an anchoring point from 0 (not perceived) to 10 (very high intensity). Additionally, the overall sensory quality (OSQ) was determined using a 5-point scoring scale ranging from 1 (very poor quality) to 5 (very good quality) based on the DGF Standard [26]. The quality level and characteristics used to determine the OSQ values of the oils are presented in Table 2.

The industrial laboratories used the 5-point quality scale to determine the OSQ values to check if oils meet the product specification requirements. In this study, we based our assessment on internal industry requirements, where the minimum acceptable scores for fresh cold-pressed and stored oils were 4.0 and 3.5, respectively.

The oil samples were coded with three-digit code numbers and served at room temperature in the amount of 80 mL in a blue jar. The panelists were instructed to clean their palates by rinsing their mouths with a weak warm black tea or eating a piece of apple. All sensory sessions were conducted in sensory laboratory designs according to ISO 8586:1 [28] and ISO 8586:2 [29].

### 2.10. Statistical Analysis

All measurements were conducted in triplicate, while the AC results were taken in five repetitions. All measurements were reported as means ± standard deviation (SD) and were processed using an analysis of variance (ANOVA) and post hoc Duncan test to determine the differences among means. A probability of *p* > 0.05 was deemed significant. The Pearson correlation and principal component analyses (PCA) were performed using Statistica (Windows software package, version 8.0; StatSoft Inc., Tulsa, OK, USA), while Fizz software (Biosystems, Courtenon, France) was applied for the collection of all sensory data.

## 3. Results and Discussion

### 3.1. Tocopherol and Phytosterol Contents in Cold-Pressed Black Cumin Oils

It is well known that tocopherols exhibit potent antioxidant properties due to their ability to donate phenolic hydrogen. Therefore, these lipophilic antioxidants are crucial for the protection of unsaturated fatty acids against peroxidation. In addition, the tocopherol profile is an important indicator of the authenticity of vegetable oils.

The results of the qualitative and quantitative analyses of tocopherols in CPBCOs are presented in Table 3.

In the studied CPBCO samples, the total tocopherol content (TTC) values ranged from 1.9 to 42.3 mg/100 g (Table 3). The highest TTC (42.3 mg/100 g) for CPBCO4 indicates pressing this oil from an entirely different seed variety or the adulteration of this oil with another vegetable oil.

For comparison, the total concentrations of tocopherols in black cumin oils pressed from the seeds grown in Macedonia, Turkey, and Egypt were 4.5 mg/100 g, 6.5 mg/100 g and 34.0 mg/100 g, respectively [6,30,31].

Concerning the tocopherol composition of the studied oils, six samples of CPBCO2-CPBCO7 (except CPBCO1) contained high percentages of the two homologous α- + γ-tocopherols varying from 94% up to 100%. A somewhat lower sum of α- + γ-tocopherol levels (57–85%) in cold-pressed and extracted black cumin oils from different countries was observed by other authors [6,8,30,31]. However, a similar percentage of α- + γ-tocopherols (98.7%) for black cumin oil extracted with chloroform–methanol from a local *Nigella sativa* L. seed variety was reported by Hassanein et al. [32].

It is noteworthy that among all analyzed samples, CPBCO4 had the lowest amount of α-tocopherol (15% of the total tocopherol content) and the highest level of γ-tocopherol (83% of the total tocopherol content). Additionally, the CPBCO1 sample had the lowest level of sum α- + γ-tocopherols (66% of total tocopherol content), whereas this oil contained the highest amount of β-tocopherol (2.9 mg/100 g). For comparison, CPBCO6 and CPBCO7 had significantly (*p* < 0.05) lower concentrations of β-tocopherol (0.4 and 0.2 mg/100 g, respectively). Interestingly, similar levels of δ-tocopherol (0.6–0.7 mg/100 g) were found only in two samples, CPBCO1 and CPBCO4 (Duncan test, *p* > 0.05, Table 3).

The significant differences indicated by the Duncan test (*p* < 0.05) for the tocopherol composition and concentration results for seven commercial CPBCOs can be explained by the influences of genetic, agronomic, environmental (seed varieties, seed growing location, etc.), and technological factors [6,8,30,31,32].

It is evident that the TTC values in all studied CPBCOs were lower than the tocopherol levels in oils pressed from other seeds, such as sunflower (28.1–383.0 mg/100 g), rapeseed (41.9–71.4 mg/100 g), camelina (97.2 mg/100 g), flaxseed (41.0–58.9 mg/100 g), hemp (69.3 mg/100 g), sesame (52.1 mg/100 g), pumpkin (29.1 mg/100 g), walnut (42.3 mg/100 g), rosehip (103.6 mg/100 g), and milk thistle (26.2 mg/100 g) [6,30,33].

Moreover, sterols are bioactive compounds that naturally occur in cold-pressed oils. They have a tendency to lower LDL cholesterol levels in the blood, reducing the risk of cardiovascular diseases. Apart from this hypocholesterolemic function, these phytochemicals and their derivatives can also be potent antioxidants [34]. Their antioxidant activities can be attributed to the formation of an allylic free radical and its isomerization to other relatively stable free radicals. In addition, the sterol composition can be considered as an important parameter for determining the adulteration or authenticity of cold-pressed oils due to each seed variety having a specific sterol profile.

It is noteworthy that the sterol compositions of the analyzed CPBCOs from the seven different manufacturers differed significantly (Duncan test, *p* < 0.05, Table 3). The total concentration of phytosterols (TSC) in CPBCO samples varied from 159 to 372 mg/100 g. As seen in Table 3, β-sitosterol was the most predominant sterol in all oil samples, followed by Δ5-avenasterol, campesterol, and brassicasterol. CPBCO4 and CPBCO2 revealed the highest and lowest β-sitosterol amounts, which were 182 and 81 mg/100 g, respectively. However, insignificant differences (*p* > 0.05) in this sterol concentrations were observed between the CPBCO3, CPBCO6, and CPBCO7 samples. The determined high β-sitosterol (48.9–56.1%) was in close agreement with those for black cumin oils from different origins (32.3–59.1%), as reported by other authors [8,30,31,32].

However, the Δ5-avenasterol and campesterol concentrations were similar for all studied oils (except CPBCO4). In addition, brassicasterol was found only in three oil samples (CPBCO1, CPBCO4, and CPBCO6), while a significantly higher brassicasterol content was present in CPBCO4 (Duncan test, *p* < 0.05, Table 3).

It can be noted that the sterol composition of CPBCO4 was not typical for black cumin oils. The high amounts of β-sitosterol, campesterol, and brassicasterol found in CPBCO4 suggest that it was adulterated with cold-pressed linseed oil.

### 3.2. Antioxidant Capacity of Cold-Pressed Black Cumin Oils

The AC results for the seven commercial CPBCOs are summarized in Table 4.

The CPBCO samples had their antioxidative properties confirmed by four in vitro antioxidant assays, namely radical scavenging (ABTS, DPPH) and reducing power (CUPRAC, FC) methods. It is noteworthy that the AC results measured using different analytical methods differed significantly (*p* < 0.05). The different mechanisms of the used analytical methods—namely single electron transfer (SET), hydrogen atom transfer (HAT), or a combination of both—probably caused these discrepancies between the AC results. It can be noted that the CUPRAC and ABTS values of the CPBCOs were higher than the DPPH and FC results. This can be explained by the fact that CUPRAC and ABTS are reactive with both hydrophilic and hydrophobic antioxidants. However, the DPPH test only allows the determination of antioxidants that can quench the purple DPPH free radicals in the alcoholic solution by providing hydrogen atoms or via electron donation and conversion to the yellow-colored non-radical form (DPPH-H). In contrast, the FC method is performed for the analysis of phenolic antioxidants capable of reducing a mixture of phosphomolybdic/phosphotungstic acid complexes in an alkaline medium, yielding a blue-colored product. Unfortunately, some non-phenolic compounds (reducing sugars and amino acids) with an FC-reductive ability can overestimate the AC values determined by FC assays [35].

The AC variability was visible between the different CPBCO samples (Duncan test, *p* < 0.05, Table 4). Definitely, the CPBCO2 had the highest AC values determined by all analytical methods (DPPH = 790.1 μmol TE/100 g, ABTS = 1465.0 μmol TE/100 g, CUPRAC = 19,823.3 μmol TE/100 g, FC = 643.7 μmol TE/100 g). The AC (except by CUPRAC value) for CPBCO2 was around four times higher than the AC of the oil with the lowest antioxidant potential (Table 4). Moreover, CPBCO5 had high radical scavenging activity against ABTS and DPPH radicals, whereas the reducing ability of CPBCO1 was found to be high in both CUPRAC and FC methods.

The Duncan test indicated that insignificant differences (*p* > 0.05) in DPPH and FC results were observed between the CPBCO6 and CPBCO7, while the CPBCO1 and CPBCO7 had similar ABTS values. As can be seen, the mean CUPRAC results significantly differed (*p* < 0.05) from each other, but the CPBCO4 and CPBCO5 samples revealed the same power to reduce Mo(VI) to Mo(V), with the subsequent formation of a green phosphate/Mo(V) complex.

The variability in AC between different the oils can be explained by genetic (seed varieties), agronomic (plant growing conditions), and technological factors, as well as the storage conditions and time. The agronomic influence on the antioxidant properties of crude oils cold-pressed from *Nigella* *sativa* L. seed varieties from Tunisia and Iran was confirmed by Cheikh-Rouhou and co-workers [5]. The polyphenol content in the oil from Tunisian variety was lower (245 mg gallic acid (GA)/kg) than the total phenolic content (TPC) in the oil from Iran (309 mg GA/kg).

The extraction technique affected the amounts of antioxidants in oils from *Nigella sativa* L. seeds. The TPC was the highest in cold-pressed black cumin oil (36.05 mg GA/kg), followed by Soxhlet-extracted oil (21.44 mg GA/kg), and was the lowest in the oil obtained from black cumin seeds using microwave-assisted extraction (15.19 mg GA/kg) [31]. However, the cold-pressed oil from Nigella seeds was a poorer source of phenolic compounds (TPC = 94.40 mg GA/100 mL), and had lower antiradical activity as determined by the DPPH method (IC_50_ = 2.30 mg/mL) and lower reducing ability as analyzed via the FRAP assay (329 mmol/100 mL) than the oil extracted using supercritical fluid extraction (TPC = 160.51 mg GA/100 mL, IC_50_ = 1.58 mg/mL, 538.67 mmol/100 mL) [36].

However, among the different cold-pressed oils (flaxseed, walnut, rapeseed, pumpkin, evening primrose, black cumin), the black cumin oil exhibited the highest lipid-soluble antioxidant capacity as determined by photochemiluminescence (7.7 mM Trolox/L oil) and DPPH (1.2 mM TE/L oil) assays [37]. The antiradical properties (DPPH = 1.85–3.18 mM TE/kg and 76.43–83.52 μmol/100 μmol) and TPC contents (115.9–119.2 mg ferulic acid/100 g and 1.02–1.40 mg GA/g) also differed for the cold-pressed black cumin oils analyzed by Symoniuk et al. [11] and Lutterodt et al. [9].

These differences between the AC and TPC results for black cumin oils observed by other authors can be explained by the use of various types of raw material, the country of origin, the cultivation conditions, the oil extraction techniques, and the applied analytical methods based on different reaction mechanisms.

### 3.3. Oxidative Stability and Quality of Cold-Pressed Black Cumin Oils

#### 3.3.1. Oxidative Stability

The oxidative stability of CPBCO samples was analyzed using the Rancimat method, and the obtained results were expressed via the induction period (IP). It is known that the IP is associated with the oil shelf life. The more extended the IP, the higher the stability against oil oxidation over time. The IP values for the studied CPBCO samples ranged between 8.31 and 37.34 h (Table 5).

As can be seen, the IP values of the examined oils differed significantly (Duncan test, *p* < 0.05, Table 5). The differences were probably related to the various cultivars used and quality levels of seeds, the different technological processes, and the country of *Nigella sativa* L. seed origin, as well as the amounts of natural antioxidants and profiles of fatty acids [5]. The highest significant oxidative stability was found for CPBCO2, and then decreased in the following order: CPBCO2 > CPBCO5 > CPBCO1 > CPBCO4 > CPBCO3 > CPBCO7 > CPBCO6 (Table 5). As expected, in the same order, the examined oils lost their radical scavenging activity, as measured using the DPPH and ABTS methods (Table 4). It is noteworthy that the low IP values for CPBCO6 (8.31 h), CPBCO7 (9.60 h), and CPBCO3 (10.14 h) indicated the low antioxidant potential of these oils, as analyzed using different analytical methods (Table 4 and Table 5).

Similar IP values that ranged between 12.00–50.33 h at 100 °C and 76.37–157.58 h at 80 °C for black cumin oils were found by other authors [5,9,11]. The higher oxidative stability of black cumin oils (13.45–38.22 h) compared to other cold-pressed oils (IP = 3.67–13.98 h for linseed, camelina, evening primrose, hempseed, poppy, and rapeseed oils) can be explained by the different fatty acid compositions and strong antioxidants such as thymoquinone [9,11,38].

#### 3.3.2. Amounts of Primary Oxidation Products and Free Fatty Acids

The CPBCO samples were evaluated for severity in oxidation and hydrolysis using respectively the PV and AV, as shown in Table 5. The PV and AV are regular indices of quality control in oils. The Duncan test indicated that the overall intensity levels of the primary oxidation products (PV = 21.36–123.77 meq O_2_/kg) were significantly different (*p* < 0.05) between the investigated oils. The amount of hydroperoxides in each CPBCO was higher than permitted by legal requirements (PV = 15 meq O_2_/kg for cold-pressed oils) [39]. Unexpectedly, the formation of hydroperoxides in the tested CPBCOs increased almost linearly (correlation coefficient, r = 0.9064) with the increase in IP values. This can be explained by the fact that lipid oxidation is a multifactorial phenomenon that depends on a number of variables.

For comparison, two cold-pressed black cumin oils and oils extracted from two Nigella seeds having an Iranian and Tunisian origin with similar PV (1.03–1.56 meq O_2_/kg and 4.35–5.65 meq O_2_/kg, respectively) showed different IP (13.45–38.22 h and 12.00–50.33 h, respectively) [5,11]. In contrast, black cumin oils obtained using microwave-assisted extraction and Soxhlet extraction with comparable PV (21.45 and 25.23 meq O_2_/kg) revealed similar IP (18.46 and 19.62 h) [31].

The tested CPBCO samples with high PV results (50.48–123.77 meq O_2_/kg) had low AV values (6.40–11.84 mg KOH/g). In contrast, the oils with lower amounts of hydroperoxides (PV = 21.36–23.17 meq O_2_/kg) and shorter IP results (8.31–10.14 h) demonstrated a higher hydrolysis degree (AV = 20.92–22.02 mg KOH/g). It is noteworthy that the AV results (6.40–22.02 mg KOH/g) of the studied CPBCOs exceeded the level recommended by the Codex Alimentarius Commission (AV = 4.0 mg KOH/g) for cold-pressed oils [39]. In our investigation, the CPBCO samples showed significantly higher acidity compared to the AV results (0.23 and 0.35 mg KOH/g) for cold-pressed black cumin oils obtained by Symoniuk et al. [11].

The obtained PV and AV results suggest that none of the assessed CPBCO samples were of acceptable oxidative or hydrolytic status, meaning they could potentially pose a health risk to consumers. In this regard, Gotoh and Wada [40] reported that oxidized fats and oils with PVs at a level of about 100 meq O_2_/kg could be neurotoxic. Furthermore, the presence of these oxidized compounds affected the sensory quality of the analyzed oils.

The estimated oxidation and hydrolytic status of the investigated oils available for retail indicated the use of low-quality raw seeds and/or inappropriate oil extraction and preservation.

#### 3.3.3. Water and Volatile Matter Contents

The water and volatile compound (WVC) levels, some of basic quality parameters for vegetable oils, were in the range of 0.05–0.26% (Table 5). As can be seen, the WVC results (0.05–0.06%) for the five CPBCO samples available for retail did not differ significantly (Duncan test, *p* > 0.05, Table 5). According to the Codex Alimentarius [39], the volatile matter of cold-pressed oils should not exceed 0.20%. The amounts of water and volatiles in only one sample, CPBCO4, were somewhat higher (0.26%) than the prescribed limit in the Codex Alimentarius [39]. It is well known that the water excess in the oil can influence its stability through triacylglycerol hydrolysis. The higher amount of water in the oil may be the result of excessive seed humidity or technological processes.

For comparison, a lower water content (0.03%) in commercial cold-pressed black cumin oil was found by Mikołajczak et al. [41].

#### 3.3.4. Polycyclic Aromatic Hydrocarbons Content

All investigated samples were analyzed for carcinogenic compounds, namely PAHs, as it is known that black cumin seeds are often dried with a conventional method where the air used for drying is heated by charcoal or gasoline.

Unfortunately, the Σ4PAHs results for the four CPBCO samples ranged between 16.09 and 46.68 μg/kg (Table 5) and exceeded the legal limit of 10 μg/kg [42]. Moreover, the level of B(a)P in the CPBCO2 (3.76 μg/kg) was significantly higher than that recommended by the European Commission [42] for foodstuffs (2 μg/kg). Interestingly, the B(a)P values of all studied oils differed significantly (*p* < 0.05), while CPBCO3, CPBCO5, and CPBCO7 showed similar B(b)F contents (Duncan test, *p* > 0.05, Table 5). Badary et al. [43] reported that thymoquinone, which is present in all black cumin seeds and oils, can reduce B(a)P-induced forestomach carcinogenesis.

It is evident that the content of Σ4PAHs was the highest in CPBCO2 (46.68 µg/kg) due to the presence of the highest amounts of B(a)P (3.76 µg/kg), B(a)A (36.98 µg/kg) and B(b)F (3.14 µg/kg) in this oil. It is noteworthy that the Σ4PAHs value for CPBCO2 was approximately 10 times higher compared to CPBCO3 containing the lowest amounts of these four carcinogenic compounds (Σ4PAHs = 4.48 µg/kg). However, the Duncan test indicated that Chry concentrations in CPBCO2 and CPBCO7 were similar (*p* > 0.05, Table 5). Insignificant differences (*p* > 0.05) in results of B(a)A concentration were observed between oil samples CPBCO3, CPBCO6, and CPBCO7 (3.20–4.00 µg/kg), as well as CPBCO1, CPBCO4, and CPBCO5 (14.48–16.47 µg/kg), respectively.

The high levels of PAHs in all investigated oil samples and a lack of professional references for PAH analyses in black cumin oils indicate it is not a well-researched topic and should be explored for human and livestock health.

### 3.4. Fatty Acid Compositions of Cold-Pressed Black Cumin Oils

The fatty acid compositions of the investigated CPBCO samples available on the Polish market are presented in Table 6. It is noteworthy that most studied oils had the typical fatty acid profile for black cumin oil reported by other authors [5,6,8,9,11,30,31,32,37,38,41,44]. The percentages of fatty acids cannot be referenced to the Codex Alimentarius, the recognized standard for vegetable oils, because black cumin oil and seeds belong to the novel food category according to the European Commission Novel Food Catalogue; thus, these values are not present in this document. As can be seen, all CPBCO samples contained moderately low amounts of saturated fatty acids (SAFA = 12.14–16.76%) and moderate levels of monounsaturated fatty acids (MUFA = 24.49–31.71%), with the predominant component being oleic acid (C18:1, omega-9 = 23.89–31.01%). It is known that MUFA, among all unsaturated fatty acids, are the most resistant to lipid oxidation, and oleic acid plays a major role in the prevention of cardiovascular diseases. However, the polyunsaturated fatty acids (PUFA), ranging between 55.27 and 59.27%, predominantly linoleic acid (C18:2, omega-6 = 32.80–59.08%), are much more prone to lipid oxidation, but on the other hand are supportive of the human cardiovascular and immune systems [44].

All investigated samples contained summed unsaturated fatty acids in the range of 83.11–87.65%, allowing the recognition of black cumin oil as a healthy fat in general.

Surprisingly, the α-linolenic acid content (C18:3, omega-3 = 23.33%) and the C18:1 percentage (31.01%) in the CPBCO4 sample were significantly higher than in all other investigated samples and were not typical for the usual fatty acid profile of black cumin oil (Duncan test, *p* < 0.05, Table 6). However, the lowest concentrations of C18:2 (32.80%) and C16:0 (8.27%) were determined in this oil. This was probably due to the high-linolenic black cumin seed cultivar used for oil production or the adulteration of this oil with other omega-3 and omega-9 fatty acid-rich oils. Similar amounts of C16:0 (8.70%) and C18:2 (34.20%) were found in cumin seed oil by Ramadan et al. [44].

### 3.5. Sensory Analysis of Cold-Pressed Black Cumin Oils

To the best of our knowledge, the sensory characteristics and sensory quality of oils cold-pressed from black cumin seeds have not been reported. In the literature, we have only found overviews on the chemical composition, nutritional properties, or influence of black cumin on the sensory characteristics of mayonnaise [45]. Bendini et al. [13] and Wroniak et al. [14] employed the QDA methodology to describe only the sensory profiles of cold-pressed sunflower oils and cold-pressed rapeseed oils, respectively.

The sensory quality and attribute intensity data of the studied CPBCO samples are listed in Table 1; Table 2.

**Figure 1:**
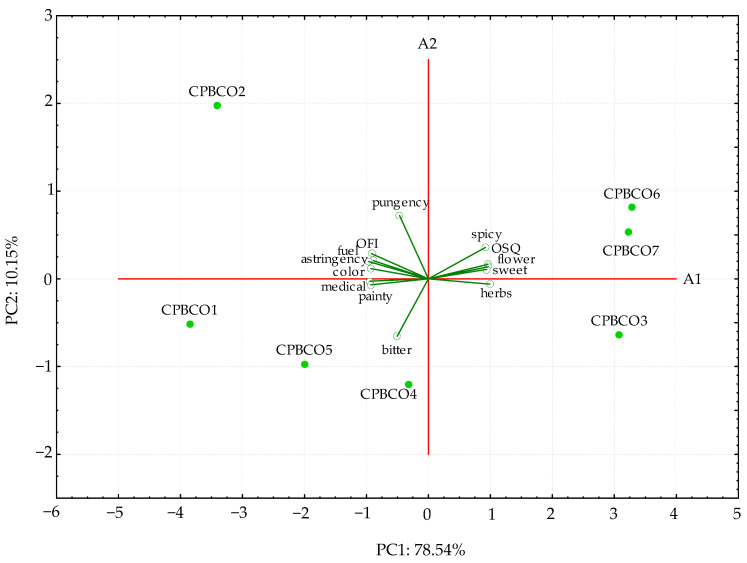
Biplot for PC1 and PC2 scores generated by the PCA of 12 attributes and OSQ for cold-pressed black cumin oils.

From the sensory quality scores, the analyzed samples can be divided into the acceptance sensory quality group (OSQ > 4.0 for CPCBO3, CPBCO6, and CPBCO7) and the unacceptance group (OSQ < 3.5 for CPBCO1, CPBCO2, CPBCO4, and CPBCO5). The PCA applied to all samples involved the mean attributes scores presented in Table 7, which were used to interpret and correlate data from 12 attributes and OSQ measured on the seven oils (Figure 1).

The first two principal components accounted for 88.69% (PC1 = 78.54% and PC2 = 10.15%, respectively) of the variability in the data set. The PCA sensory map (Figure 1) depicted that three samples (CPBCO3, CPBCO6, and CPBCO7) located on the right side of the PCA map were characterized by good sensory quality scores, as presented by the use of typical flavor descriptors (herb-like, flower-like, spicy-like and sweet-taste) for oils cold-pressed from black cumin seeds. However, some oil samples (CPBCO1, CPBCO2, CPBCO4, and CPBCO5) had poor sensory quality scores (score below 3.5 on a 5-pointing scale) with negative flavor descriptors (fuel-like, medical-like) and were situated on the left side of the PCA map. As shown in Figure 1, the CPBCO2 sample characterized by very high mouth feeling scores, mainly for astringency and pungency (about 9 points on the 10 cm scale), created an evidently distinct cluster. Furthermore, the CPBCO4 sample having a brighter (light-yellow) color and the highest bitterness perception indicated that it might be adulterated with cold-pressed linseed oil. According to Brühl et al. [46], freshly pressed linseed oil provides a delicate nutty flavor, and a lingering bitter off-taste develops upon storage at room temperature.

Nevertheless, high positive correlations were found between the OSQ and flavor attributes such as herb-like (r = 0.9462), flower-like (r = 0.9668), spicy-like (r = 0.9440), and sweet taste (r = 0.9273). However, negative correlations were observed between the OSQ and attributes such as medical-like (r = −0.9464) and fuel-like (r = −0.8642), and mouth feeling attributes such as astringency (r = −0.8724) and painty (r = −0.8880). As can be seen in Table 7, all oil samples presented high scores in terms of the OFI and were positively correlated with the color intensity (r = 0.9587). Generally, in all studied samples, the pungency, astringency, and bitter taste intensities were perceived in medium–high scores. An intense taste with the presence of bitter notes associated with pungent and astringent mouth feeling might be due to bioactive compounds such as polyphenols [47]. Different factors might contribute to off-flavors in cold-pressed oils, such as incorrect storage conditions causing the oxidative degradation of the oil, the presence of impurities in the raw material, or even the use of improper processing technology.

### 3.6. Principal Component Analysis on the Entire Set of Chemical and Sensory Data

**Figure 2:**
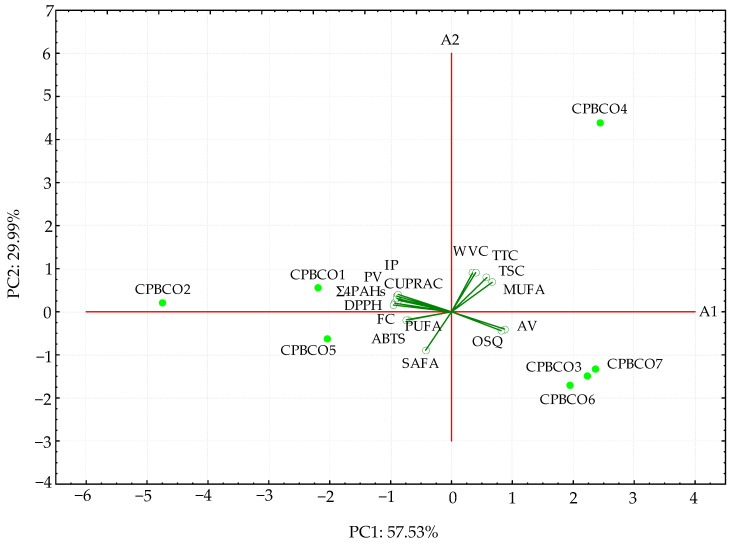
Biplot of scores and loadings of data obtained for chemical and overall sensory characteristics of the seven cold-pressed black cumin oils.

The PCA was applied to compare the multidimensional chemical and sensory quality levels of the seven CPBCO samples. The PCA model was applied to all data to determine the most important variables that explain the relationships between the investigated oils and to identify any group patterns. The PCA model retained two principal components (PC1 and PC2), which gave eigenvalues greater than 1.00 (8.63 and 4.50, respectively) and explained 87.52% of the total variability. Therefore, only the first two PCs were used to understand the similarities or dissimilarities of the CPBCO samples available for retail, as illustrated in Figure 2. The PC1 was inversely correlated with IP (−0.9012), PV (−0.9331), ∑4PAHs (−0.8727), PUFA (−0.7004), and all AC (−0.7389–−0.9534) variables, whereas PC2 was highly correlated with the moisture content and individual antioxidants (0.9027, 0.9097, and 0.7962 for WVC, TTC, and TSC, respectively).

It is noteworthy that CPBCO3, CPBCO4, CPBCO6, and CPBCO7, with high OSQ and AV scores, as well as low antioxidant properties were located to the right in the score biplot and had positive values for PC1, while the three oils CPBCO1, CPBCO2, and CPBCO5, with the highest oxidative stability (IP), hydroperoxide content (PV), and antioxidant potential (DPPH, ABTS, CUPRAC, and FC values), were situated to the left in the diagram and had negative values for PC1. However, the samples CPBCO3, CPBCO5, CPBCO6, and CPBCO7, with moderate concentrations of total phytosterols (TSC = 201–222 mg/100 g) and tocopherols (TTC = 3.9–7.2 mg/100 g), were located under the A1 axis.

It should be noted that the studied oils fell into three distinct groups (Figure 2).

The CPBCO4 with the longest distance from the other oils revealed the highest contents of tocopherols (TTC), phytosterols (TSC), MUFAs, and water and volatiles (WVC). Three oils (CPBCO3, CPBCO6 and CPBCO 7) with the highest sensory quality (OSQ) and free fatty acid (AV) contents but the lowest amounts of peroxides (PV), ∑4PAHs, and antioxidant potential as determined by different analytical assays (DPPH, ABTS, CUPRAC, and FC) created an evidently distinct cluster. Moreover, three oil samples (CPBCO1, CPBCO2, and CPBCO5) with the highest oxidative stability (IP), antioxidant properties, PV results, and ∑4PAHs, as well as the lowest AV and sensory quality, were separated from the other investigated oils. Additionally, the oils with the highest concentrations of the primary oxidation products were characterized by poor sensory quality.

Regarding the influence of impurities on the sensory profile, Wroniak et al. [14] reported a negative correlation between the level of contaminants in cold-pressed rapeseed oil and the sensory quality with the presence of off-flavors.

**Figure 3:**
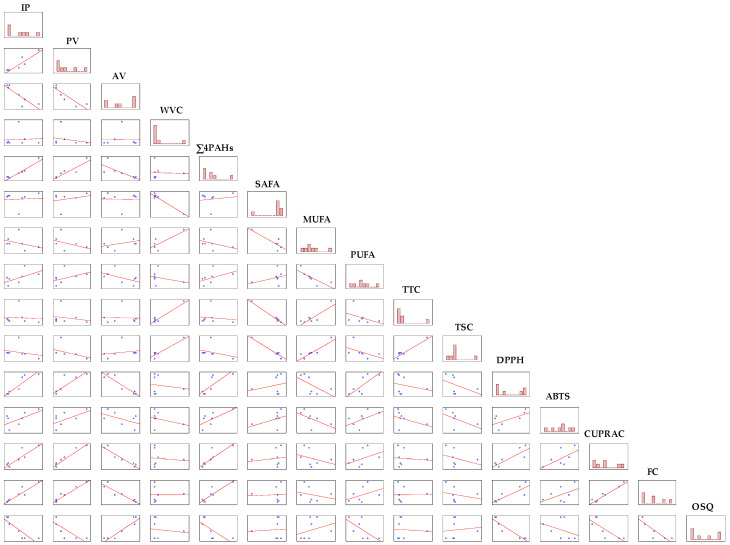
Correlation matrix between the chemical parameters and overall sensory quality levels of cold-pressed black cumin oils.

The positive and negative correlations between the chemical and sensory characteristics of the seven commercial CPBCO samples are presented as a correlation matrix in Figure 3.

Unfortunately, the OSQ of the discussed oils was significantly negatively correlated with their antioxidant potential (r = −0.8107–−0.9325, *p* = 0.0022–0.027), oxidative stability (−0.8746, *p* = 0.010), and peroxide content (r = −0.7818, *p* = 0.038), but positively associated with the level of free fatty acids (r = 0.9314, *p* = 0.0023). Moreover, there were significant positive correlations between the AC results obtained using different analytical methods (r = 0.8522–0.9732, *p* = 0.00022–0.014), AC and IP (r = 0.8842–0.9267, *p* = 0.0027–0.0082), AC and PV (r = 0.8542–0.9734, *p* = 0.00022–0.014), AC and Σ4PAHs (r = 0.8014–0.8940, *p* = 0.0066–0.030), Σ4PAHs and IP (r = 0.9597, *p* = 0.00061), and TTC and TSC (r = 0.9719, *p* = 0.00025). However, significant negative correlations between AC and AV (r = −0.9200–−0.9521, *p* = 0.00094–0.0033), SAFA and MUFA (r = −0.8569, *p* = 0.014), MUFA and PUFA (r = −0.7939, *p* = 0.033), SAFA and TTC (r = −0.9939, *p* = 0.000006), and SAFA and TSC (r = −0.9823, *p* = 0.00008) for CPBCOs were observed.

Unexpectedly, TTC and TSC did not correlate with AC, as determined by four analytical methods (r = −0.4238–0.0060, *p* = 0.3433–0.9897 for TTC and AC; r = −0.2239–−0.5376, *p* = 0.2133–0.6293 for TSC and AC). These low negative correlation coefficients suggest that the antioxidant properties of the oils may be due to more hydrophilic compounds such as polyphenols in the prepared extracts or to the synergistic interactions of tocopherols and sterols with other antioxidants present in the CPBCO samples. Therefore, the TTC and TSC in the studied oils are not reliable indicators of their overall AC. The applied DPPH, ABTS, CUPRAC, and FC methods for the determination of the AC were not equivalent by virtue of their varying sensitivities to different antioxidants found in CPBCOs. The effects of the different antioxidants did not appear to be additive but were most likely governed by synergistic and antagonistic interactions among them.

## 4. Conclusions

This study was one of the first attempts to thoroughly investigate the similarities between the sensory attributes, the composition of the CPBCO samples, and their oxidative stability.

The CPBCOs (except CPBCO4) available on the Polish market had similar chemical and nutritional values. The amounts of antioxidants and fatty acid compositions of the studied samples can be considered typical for black cumin oil. The studied oils were rich sources of unsaturated fatty acids and consisted of large amounts of linoleic acid (18:2, omega-6, >50%) and oleic acid (18:1, omega-9, >20%), while a low level of SAFA (about 16%) was found. The CPBCO4 sample was probably adulterated or unintentionally contaminated with cold-pressed linseed oil. Additionally, the sensory attributes of typical black cumin oil (including bitter taste) can be similar to linseed oil’s characteristics.

The antioxidant properties determined as the AC, TTC, and TSC and the oxidative stability of the investigated oils differed significantly (*p* < 0.05) due to differences in the raw material varieties, country of cultivation, or oil age. However, the high antioxidant potential enhanced the oxidative stability of the CPBCOs and can provide potential health benefits to consumers. On the other hand, some analyzed chemical parameters indicating the oil oxidative and hydrolytic status (PV, AV) and cancerogenic contaminant content (∑4PAHs) were above the legal limits recommended by the EU food regulations and the Codex Alimentarius standards.

Moreover, the sensory quality levels of three oil samples with the perception of off-flavor attributes were unacceptable for fresh cold-pressed oils (OSQ = 2.0). All of these defects can occur due to poor raw material quality, improper storage of the seeds, or the industrial process. For the first time, a sensory evaluation of CPBCOs was conducted by applying the QDA methodology. The sensory glossary developed in this study can be helpful for the assessment of CPBCO quality.

## Figures and Tables

**Table 1:** Sensory lexicon used for descriptive analysis of cold-pressed black cumin oils.

Sensory Attributes	Definition	Reference Product	Score
OFI	The intensity of all flavor and taste attributes taken together	1—Freshly refined rapeseed oil 8—Cold-pressed black cumin oil	1; 8
Color intensity	The intensity of brown color	Brown color intensity wheel	0; 5; 10
Sweet taste	A basic taste simulated by such as sugar	Fresh roasted cold-pressed oil	9.5
Bitter taste	A basic taste simulated by such substances as quinine and caffeine	0.2% of caffeine in water or good quality of extra virgin oil	7
Herb-like flavor	The flavor reminiscent of herbs	Fresh cold-pressed black cumin oil	6
Flower-like flavor	The flavor reminiscent of fresh flowers	Freshly cold-pressed peanut oil	8.5
Medical-like flavor	The flavor reminiscent of medical, hospital, pharmacy	Cold-pressed black cumin oil stored for 24 months in room temperature	7
Fuel-like flavor	The flavor reminiscent of fuel	Bad quality cold-pressed black cumin oil	9
Spicy-like flavor	The flavor reminiscent of cumin	Cumin seed	7
Astringency	Shrinking or drying effect on the tongue surface caused by substances such as tannins	Slices of a green banana	10
Pungency	The biting sensation that can be perceived throughout the mouth cavity	A good quality extra virgin olive oil	10
Painty	The flavor reminiscent of oils such as linseed and rapeseed oils containing linolenic acid; it cannot be noted in non-linolenic acid oils such as peanut oil	Good quality canola oil aged 4–8 days at 60 °C or until PV = 10.0 meq O_2_/kg is reached	10

**Table 2:** Overall sensory quality. General characteristics of cold-pressed black cumin oils.

Score	Quality Level	Characteristic
5	Very good	Extra fresh taste characteristic for cold-pressed black cumin oil, high intensity taste with positive attributes such as herb-like, flower-like, spicy-like, and sweet taste.
4	Good	Positive tastes such as herb-like, flower-like, and sweet-like flavor at medium intensity. Slightly bitter and astringency is acceptable.
3	Fair	Negative taste attributes such as fuel-like, medical-like, and bitter taste and mouth feeling attributes such as pungency, astringency, and painty at low intensity were identified.
2	Bad	Negative taste attributes such as fuel-like, medical-like, and bitter taste and mouth feeling attributes such as pungency, astringency, and painty at moderate intensity were identified.
1	Very bad	Negative taste attributes such as fuel-like, medical-like, and bitter taste and mouth feeling attributes such as pungency, astringency, and painty at high/very high intensity were identified.

**Table 3:** The compositions and contents of tocopherols and sterols in cold-pressed black cumin oils.

Compound	Content * ± SD (mg/100 g)						
	CPBCO1	CPBCO2	CPBCO3	CPBCO4	CPBCO5	CPBCO6	CPBCO7
α-Tocopherol	6.9 ± 0.1 ^e^	0.6 ± 0.01 ^a^	3.0 ± 0.5 ^c^	6.5 ± 0.1 ^e^	1.6 ± 0.03 ^b^	5.5 ± 0.1 ^d^	3.4 ± 0.05 ^c^
β-Tocopherol	2.9 ± 0.05 ^c^	<LOD	<LOD	<LOD	<LOD	0.4 ± 0.02 ^b^	0.2 ± 0.01 ^a^
γ-Tocopherol	<LOD	1.3 ± 0.03 ^a^	1.1 ± 0.02 ^a^	35.1 ± 0.5 ^d^	2.3 ± 0.04 ^c^	1.3 ± 0.02 ^a^	1.8 ± 0.03 ^b^
δ-Tocopherol	0.6 ± 0.01 ^a^	<LOD	<LOD	0.7 ± 0.02 ^a^	<LOD	<LOD	<LOD
Total tocopherols	10.4 ± 0.1 ^e^	1.9 ± 0.03 ^a^	4.1 ± 0.05 ^b^	42.3 ± 0.5 ^f^	3.9 ± 0.05 ^b^	7.2 ± 0.01 ^d^	5.4 ± 0.06 ^c^
Brassicasterol	2 ± 0.03 ^b^	<LOD	<LOD	18 ± 0.3 ^c^	<LOD	1 ± 0.04 ^a^	<LOD
Campesterol	30 ± 0.5 ^d^	19 ± 0.3 ^a^	24 ± 0.4 ^b^	99 ± 1.5 ^e^	24 ± 0.4 ^b^	27 ± 0.4 ^c^	25 ± 0.4 ^b^
Δ5-Avenasterol	28 ± 0.4 ^a^	29 ± 0.4 ^a,b^	33 ± 0.5 ^c,d^	33 ± 0.6 ^c,d^	35 ± 0.5 ^d^	31 ± 0.7 ^b,c^	31 ± 0.5 ^b,c^
β-Sitosterol	110 ± 1.7 ^c^	81 ± 1.2 ^a^	118 ± 1.8 ^d^	182 ± 2.7 ^f^	103 ± 1.5 ^b^	121 ± 1.8 ^e^	119 ± 2.0 ^d,e^
Total phytosterols	208 ± 3 ^c^	159 ± 5 ^a^	211 ± 9 ^d^	372 ± 14 ^f^	201 ± 4 ^b^	222 ± 2 ^e^	212 ± 6 ^d^

Note: * *n* = 3; different letters (^a–f^) within the same row indicate significant differences between amounts of tocopherols and sterols in the studied oils (one-way ANOVA and Duncan test, *p* < 0.05). Abbreviations: SD—standard deviation; LOD—detection limit; CPBCO—cold-pressed black cumin oil.

**Table 4:** Antioxidant capacity levels of cold-pressed black cumin oils.

Oil Sample	Antioxidant Capacity * ± SD (µmol TE/100 g)			
	DPPH	ABTS	CUPRAC	FC
CPBCO1	677.3 ± 16.0 ^d^	971.6 ± 20.9 ^c^	17,440.6 ± 749.8 ^f^	526.7 ± 23.7 ^d^
CPBCO2	790.1 ± 11.4 ^f^	1465.0 ± 34.0 ^f^	19,823.3 ± 915.9 ^g^	643.7 ± 25.6 ^e^
CPBCO3	276.9 ± 13.5 ^b^	385.9 ± 11.4 ^a^	975.3 ± 43.7 ^a^	206.8 ± 8.4 ^b^
CPBCO4	355.8 ± 11.1 ^c^	653.9 ± 18.3 ^b^	7402.9 ± 379.0 ^d^	354.7 ± 15.3 ^c^
CPBCO5	757.3 ± 13.2 ^e^	1270.8 ± 36.8 ^e^	8932.2 ± 549.4 ^e^	337.8 ± 13.4 ^c^
CPBCO6	229.8 ± 5.3 ^a^	1098.6 ± 13.7 ^d^	2939.9 ± 413.9 ^b^	168.1 ± 12.8 ^a^
CPBCO7	226.8 ± 6.7 ^a^	993.2 ± 32.2 ^c^	4144.0 ± 377.7 ^c^	177.0 ± 9.6 ^a^

Note: * *n* = 5; different letters (^a–g^) within the same column indicate significant differences between antioxidant activity of cold-pressed black cumin oils (one-way ANOVA and Duncan test, *p* < 0.05). Abbreviations: SD—standard deviation; CPBCO—cold-pressed black cumin oil; DPPH—2,2-diphenyl-1-picrylhydrazyl method; ABTS—2,2′-azino-bis(3-ethylbenzothiazoline-6-sulfonic acid) method; CUPRAC—cupric-reducing antioxidant capacity method; FC—Folin–Ciocalteu method.

**Table 5:** Oxidative stability and quality parameters of cold-pressed black cumin oils.

Parameter	Mean Value * ± SD						
	CPBCO1	CPBCO2	CPBCO3	CPBCO4	CPBCO5	CPBCO6	CPBCO7
IP (h)	22.05 ± 0.40 ^e^	37.34 ± 0.20 ^g^	10.14 ± 0.40 ^c^	19.7 ± 0.40 ^d^	24.92 ± 0.20 ^f^	8.31 ± 0.30 ^a^	9.60 ± 0.40 ^b^
PV (meq O_2_/kg)	89.60 ± 0.01 ^f^	123.77 ± 0.56 ^g^	22.38 ± 0.01 ^b^	35.99 ± 0.01 ^d^	50.48 ± 0.02 ^e^	23.17 ± 0.02 ^c^	21.36 ± 0.05 ^a^
AV (mg KOH/g)	6.40 ± 0.06 ^a^	7.86 ± 0.02 ^b^	21.86 ± 0.08 ^f^	15.04 ± 0.08 ^d^	11.84 ± 0.09 ^c^	22.02 ± 0.08 ^g^	20.92 ± 0.10 ^e^
WVC (%)	0.05 ± 0.00 ^a^	0.05 ± 0.00 ^a^	0.05 ± 0.00 ^a^	0.26 ± 0.01 ^c^	0.08 ± 0.00 ^b^	0.05 ± 0.00 ^a^	0.06 ± 0.00 ^a^
B(a)P (µg/kg)	0.01 ± 0.00 ^a^	3.76 ± 0.05 ^g^	0.38 ± 0.01 ^b^	0.43 ± 0.01 ^c^	0.85 ± 0.00 ^e^	1.10 ± 0.02 ^f^	0.70 ± 0.03 ^d^
Chry (µg/kg)	1.89 ± 0.01 ^e^	2.79 ± 0.01 ^f^	0.35 ± 0.01 ^b^	0.76 ± 0.01 ^c^	1.79 ± 0.09 ^d^	0.06 ± 0.00 ^a^	2.80 ± 0.07 ^f^
B(a)A (µg/kg)	16.05 ± 0.21 ^b^	36.98 ± 1.63 ^c^	3.40 ± 0.01 ^a^	14.48 ± 0.01 ^b^	16.47 ± 0.42 ^b^	3.20 ± 0.18 ^a^	4.00 ± 0.34 ^a^
B(b)F (µg/kg)	0.03 ± 0.00 ^a^	3.14 ± 0.08 ^e^	0.69 ± 0.00 ^c^	0.41 ± 0.02 ^b^	0.63 ± 0.03 ^c^	1.60 ± 0.07 ^d^	0.60 ± 0.01 ^c^
∑4PAHs (µg/kg)	17.98	46.68	4.48	16.09	19.73	7.90	6.60

Note: * *n* = 3; different letters (^a–g^) within the same row indicate significant differences between oxidative stability and quality parameters of cold-pressed black cumin oils (one-way ANOVA and Duncan test, *p* < 0.05). Abbreviations: SD—standard deviation; IP—induction period; PV—peroxide value; AV—acid value; WVC—water and volatile matter content; B(a)P—benzo(a)pyrene; Chry—chrysene; B(a)A—benzo(a)anthracene; B(b)F—benzo(b)fluoranthene; Σ4PAHs—sum of four specific polycyclic aromatic hydrocarbons; CPBCO—cold-pressed black cumin oil.

**Table 6:** Fatty acid compositions of cold-pressed black cumin oils.

Fatty Acid	Content * ± SD (%)						
	CPBCO1	CPBCO2	CPBCO3	CPBCO4	CPBCO5	CPBCO6	CPBCO7
C 16:0	12.02 ± 0.17 ^b,c^	13.03 ± 0.12 ^d^	12.06 ± 0.09 ^b,c^	8.27 ± 0.05 ^a^	12.39 ± 0.17 ^c^	11.98 ± 0.12 ^b^	12.45 ± 0.18 ^c^
C 18:0	3.10 ± 0.03 ^a,b^	3.01 ± 0.04 ^a^	3.53 ± 0.05 ^c^	3.41 ± 0.04 ^c^	3.13 ± 0.09 ^a,b^	3.50 ± 0.04 ^c^	3.40 ± 0.07 ^c^
C 20:0	0.20 ± 0.01 ^a^	0.20 ± 0.00 ^a^	0.26 ± 0.01 ^c^	0.20 ± 0.01 ^a^	0.21 ± 0.00 ^a^	0.28 ± 0.01 ^d^	0.24 ± 0.00 ^b,c^
C 22:0	0.14 ± 0.00 ^c^	0.03 ± 0.00 ^a^	0.09 ± 0.00 ^b^	0.17 ± 0.01 ^c^	0.05 ± 0.00 ^a^	0.16 ± 0.00 ^c^	<LOD
ΣSAFA	15.91	16.76	16.23	12.14	16.12	16.12	16.25
C 16:1	0.21 ± 0.01 ^b^	0.24 ± 0.01 ^c^	0.18 ± 0.01 ^a^	0.17 ± 0.00 ^a^	0.20 ± 0.01 ^b^	0.18 ± 0.00 ^a^	0.20 ± 0.01 ^b^
C 18:1	26.05 ± 0.84 ^c^	24.74 ± 0.11 ^b^	26.39 ± 0.12 ^c^	31.01 ± 0.08 ^d^	23.89 ± 0.09 ^a^	26.11 ± 0.81 ^c^	25.32 ± 0.89 ^b^
C 20:1	0.41 ± 0.02 ^b,c^	0.44 ± 0.01 ^c^	0.38 ± 0.01 ^a,b^	0.30 ± 0.00 ^a^	0.40 ± 0.01 ^b^	0.34 ± 0.00 ^a^	2.89 ± 0.04 ^d^
ΣMUFA	26.62	25.37	26.95	31.71	24.49	26.64	28.41
C 18:2	57.08 ± 0.82 ^d^	57.63 ± 1.47 ^d^	56.53 ± 2.10 ^c^	32.80 ± 1.05 ^a^	59.08 ± 2.61 ^e^	56.49 ± 2.01 ^c^	55.06 ± 1.78 ^b^
C 18:3	0.34 ± 0.01 ^b^	0.19 ± 0.00 ^a^	0.25 ± 0.02 ^a^	23.33 ± 0.90 ^d^	0.26 ± 0.01 ^a^	0.75 ± 0.03 ^c^	0.29 ± 0.01 ^a^
ΣPUFA	57.20	57.74	56.69	55.94	59.27	57.03	55.27

Note: * *n* = 3; different letters (^a–e^) within the same row indicate significant differences between the percentages of fatty acids of cold-pressed black cumin oils (one-way ANOVA and Duncan test. *p* < 0.05). Abbreviations: SD—standard deviation; C 16:0—palmitic acid; C 18:0—stearic acid; C 20:0—arachidic acid; C 22:0—behenic acid; C 16:1—palmitoleic acid; C 18:1—oleic acid; C 20:1—eicosenoic acid; C 18:2—linoleic acid; C 18:3—α-linolenic acid; ΣSAFA—sum of saturated fatty acids; ΣMUFA—sum of monounsaturated fatty acids; ΣPUFA—sum of polyunsaturated fatty acids; CPBCO—cold-pressed black cumin oil.

**Table 7:** Means for the scoring of the overall sensory quality and sensory attributes.

Sensory Attribute	Mean Value * ± SD						
	CPBCO1	CPBCO2	CPBCO3	CPBCO4	CPBCO5	CPBCO6	CPBCO7
OSQ	2.0 ± 0.0 ^a^	2.0 ± 0.1 ^a^	4.0 ± 0.1 ^c^	3.0 ± 0.1 ^b^	2.0 ± 0.0 ^a^	5.0 ± 0.2 ^d^	5.0 ± 0.1 ^d^
OFI	9.0 ± 0.3 ^c^	9.0 ± 0.1 ^c^	6.0 ± 0.2 ^a^	7.2 ± 0.3 ^b^	7.1 ± 0.2 ^b^	6.5 ± 0.1 ^a^^,b^	6.3 ± 0.2 ^a^
Color intensity	9.1 ± 0.2 ^e^	8.2 ± 0.2 ^d^	6.0 ± 0.2 ^a^	7.0 ± 0.1 ^b^	7.4 ± 0.3 ^b,c^	6.5 ± 0.2 ^a^^,b^	6.5 ± 0.1 ^a^^,b^
Flavor							
Herb-like	1.3 ± 0.0 ^a^	1.1 ± 0.0 ^a^	7.8 ± 0.1 ^d,e^	5.0 ± 0.2 ^c^	2.5 ± 0.0 ^b^	8.3 ± 0.3 ^e^	7.2 ± 0.2 ^d^
Flower-like	0.0 ± 0.0 ^a^	1.0 ± 0.0 ^b^	5.6 ± 0.1 ^d^	3.0 ± 0.1 ^c^	2.5 ± 0.0 ^c^	8.3 ± 0.1 ^f^	7.2 ± 0.1 ^e^
Medical-like	8.0 ± 0.3 ^e^	7.5 ± 0.1 ^d^	3.4 ± 0.1 ^b^	4.0 ± 0.2 ^c^	7.8 ± 0.1 ^d,e^	3.0 ± 0.1 ^b^	1.7 ± 0.0 ^a^
Fuel-like	6.2 ± 0.2 ^b^	7.9 ± 0.1 ^c^	0.0 ± 0.0 ^a^	0.0 ± 0.0 ^a^	7.2 ± 0.3 ^c^	0.0 ± 0.0 ^a^	0.0 ± 0.0 ^a^
Spicy-like	1.9 ± 0.0 ^a^	2.9 ± 0.1 ^b^	4.5 ± 0.2 ^c^	2.6 ± 0.1 ^b^	2.1 ± 0.1 ^a,b^	4.7 ± 0.1 ^c,d^	5.1 ± 0.2 ^d^
Taste							
Sweet	1.1 ± 0.0 ^a^	0.9 ± 0.0 ^a^	3.7 ± 0.1 ^c^	1.5 ± 0.0 ^a^^,b^	1.2 ± 0.0 ^a^	4.3 ± 0.1 ^d^	3.1 ± 0.1 ^c^
Bitter	6.5 ± 0.2 ^c^	4.5 ± 0.1 ^b^	4.9 ± 0.1 ^b^	7.1 ± 0.3 ^d^	4.9 ± 0.1 ^b^	3.9 ± 0.1 ^a^	3.9 ± 0.2 ^a^
Mouthfeeling							
Astringency	8.5 ± 0.4 ^d^	9.0 ± 0.4 ^d^	4.6 ± 0.1 ^a^	7.0 ± 0.3 ^c^	7.1 ± 0.2 ^c^	5.5 ± 0.2 ^b^	5.0 ± 0.1 ^a^^,b^
Pungency	7.1 ± 0.1 ^b^	9.1 ± 0.1 ^c^	6.2 ± 0.2 ^a^	7.3 ± 0.1 ^b^	6.0 ± 0.3 ^a^	7.0 ± 0.1 ^b^	6.7 ± 0.3 ^a^^,b^
Painty	7.0 ± 0.3 ^e^	6.0 ± 0.1 ^d^	2.0 ± 0.0 ^a^	4.2 ± 0.1 ^c^	7.2 ± 0.2 ^e^	3.0 ± 0.1 ^b^	3.0 ± 0.0 ^b^

Note: * *n* = 20 (10 assessors × 2 repetitions); different letters (^a–f^) within the same row indicate significant differences between attributes of the studied cold-pressed black cumin oils (one-way ANOVA and Duncan test, *p* < 0.05). Abbreviations: SD—standard deviation; OSQ—overall sensory quality; OFI—overall flavor intensity; CPBCO—cold-pressed black cumin oil.

## Data Availability

All of the data is contained within the article.

## Abbreviations

ABTS	2,2′-azino-bis(3-ethylbenzothiazoline-6-sulfonic acid)
AC	antioxidant capacity
AV	acid value
ANOVA	analysis of variance
AOCS	American Oil Chemists’ Society
B(a)A	benzo(a)anthracene
B(a)P	benzo(a)pyrene
B(b)F	benzo(b)fluoranthene
Chry	chrysene
CPBCO	cold-pressed black cumin oil
CUPRAC	Cupric-reducing antioxidant capacity
DGF	German Society for Fat Science
DPPH	2,2-diphenyl-1-picrylhydrazyl
EU	European Union
FAC	fatty acid composition
FAME	fatty acid methyl esters
FC	Folin–Ciocalteu
FFA	free fatty acids
FID	flame ionization detector
FRAP	Ferric-reducing antioxidant power
GA	gallic acid
GC-FID	gas chromatograph equipped with flame ionization detector
HAT	hydrogen atom transfer
HPLC	high performance liquid chromatography
HPLC-FLD	high performance liquid chromatography with the fluorescence detector
IC_50_	half-maximal inhibitory concentration
IP	induction period
ISO	International Organization for Standardization
LDL	low-density lipoprotein
LOD	limit of detection
LOQ	limit of quantification
MUFA	monounsaturated fatty acids
OFI	overall flavor intensity
OSQ	overall sensory quality
PAH	polycyclic aromatic hydrocarbon
Σ4PAHs	sum of four specific polycyclic aromatic hydrocarbons
PC	principal component
PCA	principal component analysis
PUFA	polyunsaturated fatty acids
PV	peroxide value
QDA	quantitative descriptive analysis
SAFA	saturated fatty acids
SET	single electron transfer
SD	standard deviation
TE	Trolox equivalents
TPC	total phenolic content
TTC	total tocopherol content
TSC	total sterol content
WVC	water and volatile matter content

## References

[1] Dedebas T., Ekici L., Sagdic O. (2021). Chemical characteristics and storage stabilities of different cold-pressed seed oils. J. Food Process. Preserv..

[2] Ketenoglu O., Kiralan S.S., Kiralan M., Ozkan G., Ramadan M.F., Ramadan M.F. (2020). Cold Pressed Black Cumin (*Nigella sativa* L.) Seed Oil. Cold Pressed Oils.

[3] Kamal-Eldin A., Moreau R.A., Kamal-Eldin A. (2009). Nigella (Black Cumin) Seed Oil. Gourmet and Health-Promoting Specialty Oils.

[4] Kabir Y., Shirakawa H., Komai M. (2019). Nutritional composition of indigenous cultivar of black cumin seeds from Bangladesh. Prog. Nutr..

[5] Cheikh-Rouhou S., Besbes S., Hentati B., Blecker C., Deroanne C., Attia H. (2007). *Nigella sativa* L.: Chemical composition and physicochemical characteristics of lipid fraction. Food Chem..

[6] Ramadan M.F. (2013). Healthy blends of high linoleic sunflower oil with selected cold pressed oils: Functionality, stability and antioxidative characteristics. Ind. Crops Prod..

[7] Tulukcu E. (2011). A comparative study on fatty acid composition of black cumin obtained from different regions of Turkey, Iran and Syria. Afr. J. Agric. Res..

[8] Ramadan M.F., Mörsel J. (2004). Oxidative stability of black cumin (*Nigella sativa* L.), Coriander (*Coriandrum sativum* L.) and Niger (*Guizotia Abyssinica Cass.)* crude seed oils upon stripping. Eur. J. Lipid Sci. Technol..

[9] Lutterodt H., Luther M., Slavin M., Yin J.-J., Parry J., Gao J.-M., Yu L. (2010). Fatty acid profile, thymoquinone content, oxidative stability, and antioxidant properties of cold-pressed black cumin seed oils. LWT-Food Sci. Technol..

[10] Burits M., Bucar F. (2000). Antioxidant activity of *Nigella sativa* essential oil. Phytother. Res..

[11] Symoniuk E., Ratusz K., Ostrowska-Ligęza E., Krygier K. (2018). Impact of selected chemical characteristics of cold-pressed oils on their oxidative stability determined using the Rancimat and pressure differential scanning calorimetry method. Food Anal. Methods.

[12] Novel Food Catalogue. https://ec.europa.eu/food/safety/novel-food/novel-food-catalogue_en.

[13] Bendini A., Barbieri S., Valli E., Buchecker K., Canavari M., Toschi T.G. (2011). Quality evaluation of cold pressed sunflower oils by sensory and chemical analysis. Eur. J. Lipid Sci. Technol..

[14] Wroniak M., Rękas A., Ratusz K. (2016). Influence of impurities in raw material on sensory and physicochemical properties of cold-pressed rapeseed oil produced from conventionally and ecologically grown seeds. Acta Sci. Pol. Technol. Aliment..

[15] Tauferova A., Dordevic D., Jancikova S., Tremlova B., Kulawik P. (2021). Fortified cold-pressed oils: The effect on sensory quality and functional properties. Separations.

[16] (2016). Animal and Vegetable Fats and Oils-Determination of Tocopherol and Tocotrienol Contents by High Performance Liquid Chromatography.

[17] (2014). Animal and Vegetable Fats and Oils-Determination of Individual and Total Sterols Contents-Gas Chromatographic Method-Part 1.

[18] Szydłowska-Czerniak A., Tułodziecka A., Momot M., Stawicka B. (2019). Physicochemical, antioxidative, and sensory properties of refined rapeseed oils. J. Am. Oil Chem. Soc..

[19] AOCS (2017). Official Method Cd 12b-92: Oil stability index. Official Method and Recommended Practices of the American Oil Chemist’s Society.

[20] (2010). Animal and Vegetable Fats and Oils-Determination of Peroxide Value-Potentiometric End-Point Determination.

[21] (1996). Animal and Vegetable Fats and Oils-Determination of Acid Value and Acidity.

[22] (1996). Animal and Vegetable Fats and Oils-Determination of Moisture and Volatile Matter Content.

[23] (1996). Animal and Vegetable Fats and Oils-Analysis by Gas Chromatography of Methyl Esters of Fatty Acids.

[24] (2000). Animal and Vegetable Fats and Oils-Preparation of Methyl Esters of Fatty Acids.

[25] (2004). Standard Practice for Sensory Evaluation of Edible Oils and Fats.

[26] (2009). Quality Evaluation of Cold Pressed Oil. Section C-Fats.

[27] (1994). Sensory Analysis-Identification and Selection of Descriptors for Establishing a Sensory Profile by a Multi-Dimensional Approach.

[28] (1996). Sensory Analysis-General Guidance for the Selection, Training and Monitoring of Assessors-Part 1: Selected Assessors.

[29] (1996). Sensory Analysis-General Guidance for the Selection, Training and Monitoring of Assessors-Part 2: Expert Sensory Assessors.

[30] Kostadinović Veličkovska S., Brühl L., Mitrev S., Mirhosseini H., Matthäus B. (2015). Quality evaluation of cold-pressed edible oils from Macedonia. Eur. J. Lipid Sci. Technol..

[31] Kiralan M., Özkan G., Bayrak A., Ramadan M.F. (2014). Physicochemical properties and stability of black cumin (*Nigella sativa*) seed oil as affected by different extraction methods. Ind. Crops Prod..

[32] Hassanein M.M., El-Shami S.M., El-Mallah M.H. (2011). Investigation of lipids profiles of nigella, lupin and artichoke seed oils to be used as healthy oils. J. Oleo Sci..

[33] Grajzer M., Szmalcel K., Kuźmiński Ł., Witkowski M., Kulma A., Prescha A. (2020). Characteristics and antioxidant potential of cold-pressed oils—possible strategies to improve oil stability. Foods.

[34] Choe E., Min D.B. (2009). Mechanisms of antioxidants in the oxidation of foods. Compr. Rev. Food Sci. Food Saf..

[35] Gulcin İ. (2020). Antioxidants and antioxidant methods: An updated overview. Arch. Toxicol..

[36] Mohammed N.K., Abd Manap M.Y., Tan C.P., Muhialdin B.J., Alhelli A.M., Meor Hussin A.S. (2016). The effects of different extraction methods on antioxidant properties, chemical composition, and thermal behavior of black seed (*Nigella sativa* L.) oil. J. Evid. Based Complement. Altern. Med..

[37] Sielicka M., Małecka M., Purłan M. (2014). Comparison of the antioxidant capacity of lipid-soluble compounds in selected cold-pressed oils using photochemiluminescence assay (PCL) and DPPH method. Eur. J. Lipid Sci. Technol..

[38] Mazaheri Y., Torbati M., Azadmard-Damirchi S., Savage G.P. (2019). A comprehensive review of the physicochemical, quality and nutritional properties of *Nigella sativa* oil. Food Rev. Int..

[39] Codex Alimentarius. Codex Standard for Named Vegetable Oils. Codex-Stan 210-1999. Adopted in 1999. Revised in 2001, 2003, 2009, 2017, 2019. Amended in 2005, 2011, 2013, 2015, 2019, 2021. https://www.fao.org/fao-who-codexalimentarius/codex-texts/list-standards/en/.

[40] Gotoh N., Wada S. (2006). The importance of peroxide value in assessing food quality and food safety. J. Am. Oil Chem. Soc..

[41] Mikołajczak N., Tańska M., Ogrodowska D., Czaplicki S. (2022). Efficacy of canolol and guaiacol in the protection of cold-pressed oils being a dietary source linoleic acid against oxidative deterioration. Food Chem..

[42] (2011). Commission Regulation (EU) No 835/2011 of 19 August 2011 Amending Regulation (EC) No 1881/2006 as Regards Maximum Levels for Polycyclic Aromatic Hydrocarbons in Foodstuffs. https://eur-lex.europa.eu/LexUriServ/LexUriServ.do?uri=OJ:L:2011:215:0004:0008:EN:PDF.

[43] Badary O.A., Abd-Ellah M.F., El-Mahdy M.A., Salama S.A., Hamada F.M. (2007). Anticlastogenic activity of thymoquinone against benzo(a)pyrene in mice. Food Chem. Toxicol..

[44] Ramadan M.F., Asker M.M.S., Tadros M. (2012). Antiradical and antimicrobial properties of cold-pressed black cumin and cumin oils. Eur. Food Res. Technol..

[45] Ozdemir N., Kantekin-Erdogan M.N., Tat T., Tekin A. (2018). Effect of black cumin oil on the oxidative stability and sensory characteristics of mayonnaise. J. Food Sci. Technol..

[46] Brühl L., Matthäus B., Fehling E., Wiege B., Lehmann B., Luftmann H., Bergander K., Quiroga K., Scheipers A., Frank O. (2007). Identification of bitter off-taste compounds in the stored cold pressed linseed oil. J. Agric. Food Chem..

[47] Gutiérrez-Rosales F., Ríos J.J., Gómez-Rey M.L. (2003). Main polyphenols in the bitter taste of virgin olive oil. structural confirmation by on-line high-performance liquid chromatography electrospray ionization mass spectrometry. J. Agric. Food Chem..

